# Sense of personal agency towards mitigating the threat of antibiotic resistance: a focus group study with parents of children under 5 years old, conducted mid-pandemic

**DOI:** 10.12688/f1000research.128550.1

**Published:** 2022-12-12

**Authors:** Becky McCall, Andrew Hayward, Michael Wilson, Gill Forbes, Laura Shallcross

**Affiliations:** 1 Institute of Health Informatics,, University College London,, London, UK; 2 Institute of Epidemiology and Health Care,, University College London,, London, UK; 3 School of Design and Creative Arts,, Loughborough University,, Loughborough, UK; 4 Centre for Behaviour Change,, University College London,, London, UK

**Keywords:** antibiotic*; 'antibiotic resistance'; parent*; 'focus group'; GP; COVID, prevention, infection.

## Abstract

**Background.** Most antibiotic prescribing occurs in primary care, largely in children under 5 years old, and often inappropriately. This study investigated knowledge, attitudes and behaviours (KABs) towards common childhood infections, antibiotic use and antimicrobial resistance (AMR), among parents of children under 5 years old. The concept of individual sacrifice (forgoing antibiotics—a selective pressure for AMR) to mitigate future societal risk of AMR and how the COVID-19 pandemic shaped views were explored.

**Methods.** This qualitative study included three, one-hour, virtual focus groups with mothers from parenting networks across inner-city London and semi-rural England, held mid-pandemic (2020). All had ≥1 child <5 years old. The Framework Method of analysis was used. Parents' KABs towards antibiotic use/AMR formed the primary outcome, with emphases on their sense of personal agency towards mitigating the threat of AMR for society, plus how the pandemic influenced views on infection prevention and care.

**Results:** Fourteen mothers (groups of six, four, four) participated, with mixed ethnicities, education and employment status. Parent perceptions of their individual child's immediate need for antibiotics outweighed concerns for any possible future threat of AMR to society. Four key themes were identified: uncertainty around symptoms; impact of socio-cultural background on KAB; poor understanding of how antibiotics/AMR work; and opportunities within the doctor–patient dialogue to shape mindset around AMR. The pandemic influenced views across themes.

**Conclusion.** Parents prioritising their child's perceived, immediate, individual 'need' for antibiotics over any future impact of AMR on society highlights a continuing need to engage parents in how to mitigate AMR through appropriate antibiotic use, reducing threat to both their child and others. Framing point-of-care dialogue around antibiotic use/AMR in the present (versus future), drawing on pandemic insights and tailoring according to nuanced socio-cultural influences, may encourage a greater sense of personal agency towards taking action to mitigate antibiotic resistance.

## Introduction

Among the multiple stakeholders tackling the global challenge presented by antimicrobial resistance (AMR), the general public, including the parents of children aged under 5 years old, the specific population in this study, play a central role.

This study refers specifically to antibiotic resistance in the UK, where around 80% of antibiotic prescribing occurs in primary care. Data show that up to 23.1% of such prescriptions are considered inappropriate by experts. ^1^

Approximately 97% of preschool children consult a doctor at least once, most often for uncomplicated respiratory tract infections (RTIs) that mostly do not benefit from antibiotics. ^2^ This suggests significant scope for improvement in antibiotic prescribing in this age group. ^3^^,^^4^

UK data from 2013 showed that 'patient knowledge, beliefs and attitudes may drive excessive antimicrobial use', including through patient influence during consultations. ^5^ Prior to the COVID-19 pandemic, gains were starting to be made, with a 13.6% reduction in antibiotic prescribing in England between 2014 and 2018. ^6^ However, despite this progress, inappropriate use/prescribing of antibiotics persists, with 2022 data showing wide variation across practices, highlighting that continued efforts to effectively counter this are needed. ^7^^,^^8^

The past decade has seen an emphasis on improving antibiotic stewardship alongside public health campaigns to improve public understanding of AMR. ^9^^–^^11^ How this has translated into parental perceptions around antibiotics/AMR formed one strand of this study—both generally, and in the light of the COVID-19 pandemic, during which this study was conducted.

Novel communication methods including the use of personalised, educational patient information leaflets together with dialogue-oriented, rather than prescription-oriented, approaches have yielded a reduction in both antibiotic prescribing and reconsultation rates. ^12^^–^^14^

However, to help optimise the patient-centric nature of this approach, a more nuanced harnessing of the socio-cultural drivers of knowledge, attitudes and behaviours (KABs) towards antibiotic use and AMR, within the doctor–patient dialogue around AMR, may impact personal attitudes, social norms and perceived barriers to responsible antibiotic use – an articulated objective of the UK Government. ^5^^,^^15^

With respect to socio-cultural influences on KABs of AMR, this study was conducted in the middle of the most significant infectious disease pandemic for a century—COVID-19. As such, consideration was given to the influence of the pandemic on views around infectious disease prevention and management.

The fundamental concept of adopting an individual sense of personal agency, for example, foregoing an antibiotic (for non-serious infections) to help mitigate AMR for future societal benefit provided this study with a novel lens though which to understand parent perceptions. This is an often-used concept that frames AMR as a humanitarian crisis potentially leading to 10 million deaths by 2050, but this may precipitate a sense that personal action to avert this destiny is beyond an individual's reach, as echoed in another study, with parents, 'unsure as to how they could reduce antibiotic resistance themselves as the problem was part of a “much bigger” picture’. ^16^^,^^17^

This study aimed to obtain a snapshot of the perceptions and behaviours (including parent perceptions of doctor–patient interactions) around antibiotic use and AMR of parents with respect to their children, and to interpret these through the novel lenses of both a sense of personal agency towards mitigating the threat of AMR for individual as well as societal gain, both now and in the future, and, uniquely, within the setting of the COVID-19 pandemic.

## Methods

This qualitative study included three focus groups comprised of parents of children aged under 5 years old. Originally planned as an in-person activity, pandemic restrictions required an amendment to the original NHS Health Research Authority (HRA) ethical approval (REC number 19/LO/1820, HRA approval February 2020). The duration of each focus group protocol was adapted to approximately one hour, deemed optimal for a virtual focus group. ^18^^,^^19^

### Participant selection

Participants were recruited using a purposive sampling method. Two easily reachable geographical areas (originally selected for in-person groups) were chosen providing a mix of inner-city urban (Islington, London; one group), and semi-rural (Hertfordshire; two groups), as well as diversity in ethnicity, educational attainment and employment status. Leaders of preschool parenting networks (employees of Islington Borough Council and Hertfordshire Community NHS Trust) facilitated recruitment through official social networks, for example Facebook groups or weekly meetings, as well as *via* parent champions who recruited both directly and through snowball sampling until sufficient numbers were reached.

Participants were provided with participant information sheets and electronic consent forms, along with an email introducing the study. Author BM answered any questions to ensure fully informed consent was given, and participants signed and filed the consent forms electronically, and received a £20 voucher for their contribution.

### Data collection/topic guide

Parents’ basic demographic data were gathered, including employment status, ethnicity and parity. Participants were asked to respond in their capacity as parents.

The topic guide listing *a priori* themes was jointly developed by the researchers, including two with experience in clinical and public health issues related to antibiotic use/AMR (authors LS and AH). It was structured to provide a snapshot of current understanding around antibiotic use, and both the threat felt (if any), and the sense of responsibility participants held towards their children, and others, in mitigating the emergence and spread of AMR. Topics addressed infection self-care, antibiotic-seeking KABs, the nature and consequences of AMR including sense of individual agency in mitigating AMR for societal good, and COVID-19 influence on views towards infection prevention and management. After piloting by two parents of young children, slight modifications were made to enhance the lay-friendly appeal of content.

Author BM was the focus group facilitator, and only member of the research team attending the sessions. Secure and encrypted MS Teams video conferencing technology was used to collect and record the focus group data. All participants contributions were transcribed and anonymised.

(See Table 1 for a topic guide summary; see extended data for full topic guide).

**Table 1.** Summary of topic guide questions. ^20^^–^^22^

1. **Approach to treating illness (infections) in your child (including the influence of COVID-19 pandemic on infection prevention and management)** ‐ For which illness/es and symptoms would you seek healthcare professional (HCP)/doctor advice? ‐ Who and what services have you contacted *, e.g.* out of hours, walk-in centre? ‐ How would you provide home care, and over the counter advice/treatment (prior to approaching a doctor)? ‐ Have your children received recommended childhood vaccinations? ‐ Do you consider vaccination an important preventive measure against infectious disease? ‐ How has COVID-19 influenced your views towards infection prevention and management including the potential for COVID-19 vaccination (both for you and your children)? 2. **Antibiotic use: experience of your child (or you secondarily)** ‐ When did you last see an HCP/clinician in a situation when you thought your child might need an antibiotic (and either received an antibiotic or not)? ‐ Have you ever been refused antibiotics and how did that conversation play out between you and the clinician? ‐ Does your doctor ever initiate a discussion around the need for antibiotics, and, if so, does s/he refer to the downsides of antibiotic use, *e.g.* resistance or other? ‐ Have you ever sought antibiotics from somewhere other than your GP, and if so, why ( *e.g.* walk-in centre, out-of-hours centre, NHS111, online)? ‐ Do you consider that antibiotics can be harmful as well as beneficial? Expand on harms versus benefits. 3. **Antibiotic resistance and perceived threat, if any, from AMR** ‐ What do you understand by the term ‘antibiotic resistance’ (AMR)? ‐ How does antibiotic resistance develop (including any mechanistic understanding)? ‐ To what extent do you feel personal risk (or risk to your child) from a drug-resistant infection/AMR? 4. **Societal consequences of antibiotic resistance and responsibility/agency to mitigate effects** ‐ Do you believe your personal actions towards antibiotic use can influence development of AMR and have an impact on your/your child's health and that of the wider public in the long-term? (Possible parallel with potential COVID-19 vaccination to prevent community spread.) ‐ What are the direct and indirect consequences of AMR for your child as an individual but also for society in the longer-term? ‐ People talk about ‘an antibiotic crisis’, what does this mean to you? ‐ Do you think individual (personal sense of agency) or governments/big organisations should take greatest responsibility for tackling the threat of AMR?

### Data analysis

Transcripts of the recorded discussions were entered into Computer-assisted qualitative data analysis software (CAQDAS, NVivo Version 12 (RRID:SCR_014802)) to organise the data. Alternative open access software to NVivo exist for example, Taguette or RDQA.

Qualitative analysis followed the established Framework Method. This entailed a stepwise process of data familiarisation; mostly inductive analysis consisting of line-by-line coding/sub-coding (using NVivo) and grouping into categories broadly aligned (but adapted to reflect conceptual relationships between comments) with the *a priori* topics in the topic guide, and this effectively created an analytical framework. This was applied across all transcripts to compare cases (participant comments) both within focus groups and across focus groups until no new codes were generated (data saturation point). By charting the data into a ‘framework matrix’ comprising codes in columns, and cases in rows (see extended data for a table of codes and descriptions, and for an example of the framework matrix), thematic analysis was conducted. This involved the generation of sub-themes initially, and then key themes (synthesis across sub-themes). Ultimately, insights (possible unarticulated explanations) were derived from reviewing the matrix and drawing connections within and between participants and categories to facilitate higher order interpretation, according to a process of thematic analysis. ^23^^–^^26^

Coding and themes were checked for consistency and reliability with two co-authors (LS and AH) and to balance any reflexivity of BM, a mother of young children, in analysis of the data. Such similarity may be considered a bias, but also a benefit that may enhance rapport and the richness of data obtained. ^27^

Consolidated criteria for reporting qualitative research (COREQ) were followed as closely as possible in the reporting of this research. ^28^ In accordance with these guidelines, lead author BM attended training in both facilitating a focus group and conducting qualitative data analysis. BM had no relationship with participants prior to the study and the participants understood the research formed part of BM's doctoral research.

### Patient involvement

Four members of the public were consulted in designing the topic guide and provided input on content and style of questions, as well as proofing.

## Results

A total of 14 parents participated in three virtual focus groups between August and October 2020, with a topic guide adapted from the in-person to the virtual format. The adapted virtual format shortened the focus group duration and the last section on public health campaigns was insufficiently answered to warrant inclusion. Two participants dropped out: one due to technical connection issues, and one due to a hospital appointment. One other was a medical doctor and it was decided this potential participant would be too conflicted to include in this study that sought views from parents who were members of the lay public.

Most participants (10 out of 14) were aged 30–40 years and White British (nine out of 14); all were female and with at least A level or equivalent of educational attainment, and all had at least one child under five years. Most (11 out of 14) children had received antibiotics at least once: eight out of 14 from a GP, and six out of 14 from secondary care or an NHS walk-in centre (see Table 2).

**Table 2.** Demographic characteristics of parent participants.

Demographic characteristic (number participants)	Aggregated total (14 participants across all three focus groups)					
**Age (years)/n**	18–30 (1)	30–40 (9)			40+ (4)	
**Gender (female)/n**	14					
**Ethnic origin (n)**	North African (2)	Somali (1)	White British (8)	Pakistani (1)	Indian (1)	White Jewish (1)
**Number of parents with children < *or* < and >5 years old(n)**	<5 years old (12)			<5 and ≥5 years old (2)		
**Highest educational qualification (n)**	A levels/HND (2)	Tertiary qualification *e.g.* BA or BSc or HND (5)			Higher degree, *e.g.* MSc or PhD (7)	
**Occupation**	Homemaker (4); administrator (3); social worker (1); teacher (2); manager (3); solicitor (1).					
**Received antibiotics**	Yes (11)			No (3)		
**Antibiotic prescriber**	Primary care (8)	Secondary care (5)	Pharmacy (0)	Emergency room/A&E (0)	Out of hours (1)	
**Conditions antibiotics prescribed for**	Peri-oral dermatitis and conjunctivitis; chest infection; croup; daily antibiotic to prevent and treat urinary tract infections; severe rash; surface wound on the face; tonsillitis; pneumonia; nappy rash; umbilical granuloma; ear infection (4); infected finger.					

In accordance with SAGER guidelines for reporting sex and gender information in studies, this study was designed to recruit parents, without gender discrimination. Only mothers volunteered to join the focus groups.

### Themes and insights generated

The process of analysis using the Framework Method generated 13 codes, divided into four categories that emerged from the data (see extended data for a table of descriptions according to categories and codes; and a table of categories aligned by their codes and sub-codes).

Analysing each transcript according to these codes and selecting verbatim illustrative of them generated insights including drivers of KABs around AMR. Synthesis across the three focus groups generated four key themes, which are presented alongside their sub-themes, codes, and categories/ *a priori* topics in Table 3.

**Table 3.** *A priori* topics as related to codes, sub-themes, and key themes (generated by inductive analysis).

*A priori* topics (from topic guide)/categories	Codes (sourced via inductive analysis)	Sub-themes (derived after applying analytical framework to transcripts)	Key themes
**1. Approach to managing illness (suspected infections) in the child**	1. Homecare (inc. symptomatic relief/remedies); when, *i.e.* which symptoms and severity, to seek professional medical help 2. Understanding of infection control including vaccines 3. COVID affecting views of infection – causal agents, prevention and management	Sometimes a disease has no treatment—reflective of COVID at this time (summer 2020) Antibiotics are not a cure-all Bacterial vs. viral infections: each treated differently COVID—greater awareness of viral infections (compared with bacterial) Try other possible solutions/symptomatic relief before antibiotics, including riding it out Thinking it is not serious enough for GP/antibiotics Dislike and avoidance of healthcare environment—fear of (further) infection (COVID pandemic) Vulnerability to infections in some children (underlying problems), especially in light of COVID (a central message relevant to COVID) Worries (real or other) related to being the mother of a young child, especially first time Stay as healthy as possible to avoid infections (diet, exercise) Background, *e.g.* cultural and generational reasons for seeking/taking antibiotics Source antibiotics abroad and keep for when needed, *e.g.* 'in the cupboard' Changed habit, *e.g.* from frequent antibiotic use to less use Disagreement/tension with doctor over antibiotics Vaccines and infection prevention: infection control via vaccines or other to avoid infection (and consequently possible antibiotics). COVID vaccines under discussion at this point (not widely available) and some discussion reflected motivation to undergo vaccination and benefit to individual and/or society	**Key theme 1**. Uncertainty in the management of symptoms and severity of childhood infection, including when to consult a GP (possibly for antibiotics), including influence of the pandemic on views around prevention and management of infection **Key theme 2**. Background comprising cultural, social, generational and habitual factors that influence attitudes and behaviours towards antibiotic use (and, by extension, AMR)
**2. Antibiotic mode of action, use and interaction with clinician around the need for antibiotics**	4. How antibiotics work—some basic mechanistic information 5. How antibiotics are used or how we are exposed to them 6. Attitude towards self vs child 7. Patient–doctor interaction around antibiotics	Some mixed understanding but most people knew antibiotics kill bacteria, not viruses Specific or broad spectrum Broad spectrum use causes more resistance to develop Antibiotics stimulate production of antibodies to infections (possible confusion with vaccination) Test sensitivity of the bug: test sensitivities to ensure right antibiotic for right bug Antibiotics inject something into body cells, *etc.* Antibiotics needed if person is old or has co-morbidities Antibiotics overused in animals and agriculture Doubt about best course of action in the child: use may cause resistance, non-use, then infection worsens More cautious with completing course as prescribed for child (vs. for themselves as adults) Communication GP to patient: tension between following parental instinct *or* following GP advice Expectation to get antibiotic or some other type of medicine from GP Trust the doctor vs. doubt the doctor (including doctor–patient tensions) Alternatives to antibiotic prescription, *e.g.* delayed prescription; patients using leftover antibiotics Attendance out-of-hours/A&E services including under-confident first-time mothers; no GP appointments available	**Key theme 1**. Uncertainty in the management of symptoms and severity of childhood infection, including when to consult a GP (possibly for antibiotics), including influence of the pandemic on views on prevention and management of infection **Key theme 3**. Greater understanding of how antibiotics work, and how AMR develops, may impact perception of threat to the individual and society, and consequently individual sense of agency/responsibility towards mitigating AMR **Key theme 4**. Strength of the doctor–patient dialogue serves as an opportunity to effect change in knowledge, attitudes and behaviour (KABs) relating to AMR at the point-of-care
**3. Mechanisms of antibiotic resistance (bacterial AMR); personal sense of threat from AMR**	8. AMR and how it arises 9. Responsible use of antibiotics (immediate term) in order to mitigate AMR (longer-term)	Bacteria become resistant Body becomes less able to fight infection (with repeated antibiotic use) GP/HCP-led education Mechanistic explanations for AMR, *e.g.* bacteria 'adapt' Kills good bacteria in body too and makes you more susceptible to infections (cross-reference to category 2) Body (not bacteria) becomes resistant Overuse or misuse of antibiotics drives AMR HCPs/GPs pushing education Doctors changed approach to discussing antibiotic use/AMR with patients Posters and ads Cultural shift (or not) in patient expectations of receiving antibiotics Campaigns to change use of antibiotics in food production, agriculture, *etc.* (cross-reference category 4) Government guidelines, pressure to encourage more cautious approach to ABX use (cross-reference category 4) Multi-faceted approach from all to curb antibiotic use and be responsible for AMR (cross-reference category 4) Consistency in approach to messaging and campaigning (by whoever) Tailoring messages and campaigns to different demographics, *e.g.* older people or mums, *etc* (cross-reference category 4)	**Key theme 2**. Background comprising cultural, social, generational, and habitual factors that influence attitudes and behaviours towards antibiotic use (and, by extension, AMR) **Key theme 3**. Greater understanding of how antibiotics work, and how AMR develops, may impact perception of threat to the individual and society, and consequently individual sense of agency/responsibility towards mitigating AMR **Key theme 4**. Strength of the doctor–patient dialogue serves as an opportunity to effect change in knowledge, attitudes and behaviour (KABs) relating to AMR at the point-of-care
**4. Consequences of inappropriate antibiotic use: longer-term societal consequences of AMR and individual agency to mitigate effects**	10. Worries about AMR threat 11. No solution, drugs do no work 12. Concern for self from AMR and from infections 13. Concern for society from AMR's effects	Fear of no antibiotics due to resistance, no antibiotics for surgery, *etc.* Lack of antibiotics—a problem now or future Never been very worried about bacterial infections because there is a treatment—antibiotics. 'It’s the viral I’m scared of'. Disbelief that one day we may run out of working antibiotics Lack of universal consensus on how to control threat of AMR Child has chronic disease (UTIs) and needs ABX all the time Dilemma of promoting AMR or my child having serious infections Avoid antibiotics and end up sicker (personal reasons and burden on the NHS) Personal vs societal problem Treat child differently to self Real event, *e.g.* relative/friend made AMR real More pressing problems in the world Petrified of AMR Worried about uncontrollable use of ABX in food/agriculture Misuse—overuse	**Key theme 1**. Uncertainty in the management of symptoms and severity of childhood infection, including when to consult a GP (possibly for antibiotics), including influence of the pandemic on views around prevention and management of infection **Key theme 3**. Greater understanding of how antibiotics work, and how AMR develops, may impact perception of threat to the individual and society, and consequently individual sense of agency/responsibility towards mitigating AMR

Key themes 3 and 4 are most novel, while some aspects of key themes 1 and 2 are relatively new but largely reinforce findings from other studies. Table 3 shows the relationship of the original *a priori* topics (from the topic guide) to sub-themes and key themes (sub-themes and key themes were derived from inductive analysis).

**Key theme 1: Uncertainty around the management of symptoms and severity of childhood infection, including when to consult a GP (possibly for antibiotics), including the influence of the pandemic on views around prevention and management of infection.**

Most parents remarked on their uncertainty about when the severity of their child’s symptoms warranted a doctor consultation, possibly for antibiotics. Many said they home-managed symptoms for around three days before seeking medical help. Mothers with older children (compared with first-time mothers) suggested greater confidence in managing their child's illness at home for longer.

One mother recalled giving her 10-month old 'Calpol' if he had a fever, but if she felt his *‘heart rate was up and he wasn’t feeding properly’* then she would contact a doctor *.* (Focus Group (FG)2, participant (p)3)

*'I didn’t take her for a few weeks because I was like, “Oh she’s teething, she’s got a bit of a cold, she’s just gone back to nursery after lockdown”.*’ (FG2, p4)

*‘As a first-time mum, you’re a little bit more nervous and apprehensive about things, having not done it before.’* (FG2, p3)

Such comments highlight the need for improved public/patient engagement around which symptoms and severity justify contacting a GP (potentially seeking antibiotics).

Sometimes non-medical pressures are implicated—economic (employment-related) factors were alluded to.

*'It’s a bit selfish but I find it quite hard to get her to the doctor, because you have to take time off work, I’ve got to get her out of nursery.'* (FG2, p4)

The COVID-19 pandemic profoundly influenced people’s attitudes and behaviours towards infection prevention and control, with constant public health messages around handwashing and other SARS-CoV-2 mitigation measures. Community antibiotic prescriptions fell during this period. ^29^

*‘Covid has raised my awareness of infection, and actually, not everything can be treated by antibiotics, and sometimes, you’ve just got to ride things out depending on the severity of your child’s illness.’* (FG1, p6)

Vaccination, generally, was also raised as a measure to prevent infection and was particularly topical at the time, with potential COVID-19 vaccinations being debated publicly.

Table 4 lists examples of verbatim relevant to this key theme.

**Table 4.** Key theme 1 verbatim. Uncertainty in the management of symptoms and severity of childhood infection, including when to consult a GP (possibly for antibiotics), including the influence of the pandemic on views around prevention and management of infection.

• Uncertainty about when symptoms (and severity) justify GP care, and home management of symptoms. *‘I just check out the NHS website as a first point of call, if I don’t believe that the symptoms appear that serious.*’ (FG1, p5) *‘… it’s important to stay healthy, by having a good diet … I take probiotics - so vitamins, probiotics to avoid infections as well.*’ (FG1, p4) *‘With my little one I barely gave him any antibiotics. I’ve changed my approach I would say. I’ve been in the UK for 15 years now.*’ (FG3, p1) *‘… I have four children, oldest 15, youngest three, so, with something like tonsillitis, they would have to have antibiotics, but I would go to the GP’.* (FG2, p2) Another parent misinterpreted messages around AMR and avoided the GP, resulting in her child being admitted to hospital. She recalled that had she, *'had just taken the antibiotics in the first place then I [her child] wouldn’t have been*'. (FG 2, p1) • Parents were more cautious with their child than themselves (sense of child's vulnerability). *‘ … when I was prescribed the [antibiotic] cream for my child I did not hesitate, I was gonna do it.' and ‘… for my daughter I’d always finish the course.’* (FG3, p4) • Parents who took their child to an out-of-hours/walk-in/emergency centre most often received an antibiotic prescription. *‘Almost any time I ended up at out-of-hours or A&E, I got antibiotics, even if I was told they weren’t needed only a few hours earlier.’* (FG1, p3) *‘… we delayed and kept him at home for a while and then went to an out-of-hours [service], and they just instantly prescribed antibiotics. I think when it’s a chest infection, I’d rather have antibiotics to clear it up, I think because when it’s something to do with breathing …*’ (FG2, p1) • Vaccination to prevent infection was mostly supported. *‘My mum was an anti-vaxxer so I wasn’t vaccinated until I was 18 when I could make the decision myself for that. As a result of not having those vaccines, I’ve got cancer-causing HPVs and I need so many checks—I’m angry because of that, so, with [daughter’s name], she was going to have every vaccine possible, so she doesn’t have the same experiences that I’ve had.*’ (FG1, p4) *‘I’m having more fruit and veg, the same for my kids, I’m really not into having a vaccine [with respect to COVID-19], or having some antibiotics, I know that it wouldn’t resolve the problem, because we don’t know what it is, and I don’t think they know how to cure it anyway.’* (FG3, p1) • COVID-19 has heightened concerns about catching infections (SARS-CoV-2 or other). ‘ *It’s hospital but if I can do anything else before I take them to a place where other sick people go [response to COVID pandemic discussion], I’ll do it.’* (FG1, p3) ‘ *Had he been born outside of Coronavirus times, I probably wouldn’t have been as concerned about him catching infections … I wouldn’t have been as worried about it as I am now, and I think that’s the same with antibiotics.*’ (FG 3, p1)

**Key theme 2: Background including cultural, social, generational, familial and habitual factors influence KABs towards antibiotic use/AMR**

Subtle and often unarticulated factors, including generational, familial, cultural, habitual, geographical and social, often influence views towards healthcare, including antibiotic use/AMR, as well as parent/patient expectations from the doctor–patient consultation.

One mother who grew up in Somalia said they expected to receive medication upon visiting the GP, effectively as a validation of their illness, ‘ *be it antibiotic or medicine or anything*'.

Another mother said that antibiotics were habitually prescribed in her parent’s home country, France.

*‘… in France when you get sick, it’s antibiotics … . sometimes if my kids were really, really sick I would buy medication from France and bring my medication here. My mum, she sends me parcels [with antibiotics].’* (FG3, p1)

Older generations lived in a time when antibiotics were considered a cure-all.

*‘I think it’s difficult for the older generation that think that antibiotics are the answer to everything.’* (FG3, p4)

A mother from Spain said antibiotics were much more available there than in the UK, and, in her opinion, there was a lack of universal consensus around antibiotic stewardship.

*‘In the UK, antibiotics for urinary tract infections [UTIs] require antimicrobial pharmacist approval, … whereas, in Spain, they just sort of hand [them] out left, right and centre.’* (FG1, p4)

Understanding more about the socio-cultural drivers and experiences underpinning an individual's KABs towards antibiotic use may illuminate novel, more nuanced, avenues for formulating and communicating AMR messages.

Certain areas of the UK with greater socio-cultural diversity may benefit most from incorporating a more nuanced and tailored approach to the doctor–patient dialogue around antibiotics/AMR.

**Key theme 3: Greater understanding of how antibiotics work, and how AMR develops, may impact perception of threat to the individual and society, and consequently individual sense of agency/responsibility towards mitigating AMR**

Overall, lay understanding of how antibiotics and AMR work was mixed but largely limited. Most parents knew that antibiotics fight bacteria, not viruses, with one mother understanding that antibiotics can be either narrow or broad spectrum. That antibiotics can target good bacteria too was mentioned. *‘… you don’t know what’s getting killed basically …’* (FG2, p4)

*'It's a bit like the bug becomes stronger, a bit like we’re seeing with Covid and they’re talking about all these different strains, and it mutates – AMR, that’s what I understand.'* (FG2, p1)

Four participants believed that antibiotic resistance meant the body became tolerant to antibiotics.

*'Your body changes over time, something that worked for you once, might not work for you again.'* (FG1, p5)

The relatively common misunderstanding that resistance implies the body becomes tolerant to antibiotics, rather than resistance being a feature of the infecting bacteria, may be instrumental in the sense of threat individuals perceive from AMR, and may help to explain why many people see AMR as a threat not associated with them personally, but only with others who use antibiotics frequently. Some parents felt the threat of AMR was not very present in their lives today.

*‘I think it is on the risk scale but it’s not something that I worry about every day.’* (FG1, p1)

*‘I had always thought, like you said, it’s something that will happen in the future or something that’s coming but it’s not really [here now].’* (FG2, p4)

AMR is often framed as one of society's most serious global humanitarian crises, and, as such, participants were asked whether they would personally forego an antibiotic now to prevent future widescale AMR adversity for society. Despite some parents recognising this, all parents said that the immediate threat of infection to their individual child’s health took priority and that they would seek antibiotics if it promised to help their child.

*‘Do I really want him to become antibiotic resistant? But equally I don’t want him to get infections.’* (FG2, p1)

Parents reflected on securing a future free of AMR and the benefit it offered to their child in preference to society. Together, these comments suggest that focusing on benefit to the individual rather than society would potentially resonate more with parents.

Two parents highlighted that the lack of an effective treatment for COVID-19 (summer 2020) echoed the nature of AMR, whereby antibiotics are ineffective against infection.

*‘There’s a bit more awareness that not all illnesses have a definitive treatment, and that antibiotics are [not] the cure-all as some people believe.*’ (FG1, p1)

The mitigation measures taken by individuals to reduce societal spread of COVID-19, *e.g.* vaccination, isolation, masks and social-distancing, reflect the narrative pertaining to AMR where individual action now (foregoing an antibiotic) helps to avoid future adverse effects for society (emergence of AMR later).

Most parents said they would have a COVID-19 vaccination, when they became available. Of note, the groups were held mid-pandemic and the threat of COVID-19 felt real, such that the risk of future adversity (COVID-19) was considered serious enough to warrant the small inconvenience and/or discomfort of vaccination. By extension, this might highlight the value of imparting knowledge about the real and immediate threat of AMR and how to avert it when people are primed to receive such information, for example, at the point of care, when parents consult a GP with a sick child.

Table 5 lists examples of verbatim relevant to this key theme.

**Table 5.** Key theme 3 verbatim. Greater understanding of how antibiotics work, and how AMR develops, may impact perception of threat to the individual and society, and consequently individual sense of agency/responsibility towards mitigating AMR.

• There was some understanding around how antibiotics work but also a need for further clarification for most participants. ‘… *they inject something, like into the body, in a small amount so then the body can fight against it, something like this.’* (FG3, p1) *‘… some antibiotics can be … broad spectrum. They will kill more bugs than other ones …*’ (FG1, p1) • Inappropriate use of antibiotics relates to animal use as well as human. *‘...like the chicken, they always inject with antibiotics, so actually we’re taking them without realising we’re having antibiotics …*’ (FG2, p1) • A mixture of confusion and some understanding around how AMR develops *‘… because we’ve been taking the same type of medication it’s not going to have an effect on your body, because your body knows about this treatment … when you have taken them frequently, your body can build up a tolerance to them so they don’t necessarily fight off the bacteria as effectively as they might have done the first time you were prescribed them.’* (FG2, p1) *‘… if your immune system isn’t given the opportunity to work and to attack both the build-up of antigens and antibodies in your own body, that will mean you are less good at fighting other diseases in the future.*’ (FG1, p1) • There was some confusion around whether the threat posed by AMR was a real threat today, or a possible threat for the future. One parent said she was *‘terrified’,* and that AMR is *‘extremely widespread’.* Using COVID-19 as a reference, she added, ‘ *COVID-19 is bad, but I really am a lot more concerned by AMR’.* (FG1, p3) • COVID-19 highlighted to some parents that not all infections have an effective treatment. *‘I think people are a bit more aware of the untreatable diseases and ones that are viruses as opposed to other infections.’* (FG1, p1) • Threat of a current infection was more real than threat associated with any possible resistant infection in the future. One child was ill with a urinary tract infection *. ‘… it’s the lesser of two evils … I guess you’ve just got to deal with what you’ve got to deal with at the time haven’t you?’* (FG2, p4) *‘I’d be more worried about my son than I would be about myself. The way I perceive it is that it’s going to happen over time, or maybe to me when I’m older.’* (FG3, p3) *‘I’ve lived so much in a bubble where antibiotics have cured everything, for me personally, and people I know, I’ve not really thought about it.’* (FG2, p1) ‘ *I do look at it as more of a personal problem with individual bacterial resistance rather than being a kind of, do it for the community, so I take it as a personal kind of thing for me and my son.*’ (FG2, p3) • Many did not consider AMR a real and current danger. Some parents believe science will find an answer. *‘It’s not really crossed my mind that there wouldn’t be a solution to a bacterial infection.*’ (FG2, p1)

**Key theme 4. Strength of the doctor–patient dialogue serves as an opportunity to effect change in KABs relating to antibiotics/AMR at the point-of-care.**

Participant comments in this study indicate that the primary care consultation setting is conducive to a constructive interaction around the risks and benefits of antibiotics and AMR. ^12^^–^^14^ As such, these data both add value to, and reflect, prior reports of antibiotic stewardship leading to a reduction in antibiotic prescribing in primary care over recent years (excepting anomalies related to pandemic prescribing). ^6^^,^^30^ Such constructive dialogues nurture improved GP patient trust and enhanced compliance, potentially reaping short and longer-term benefit.

*‘Now they are clearer when they say it, so I trust my GP.’* (FG3, p1)

That clinicians have a valuable role to play in alleviating misunderstanding around antibiotics, and AMR was widely accepted by parents, most of whom said that they followed the doctor’s advice on antibiotic use.

*‘If they think it [an antibiotic] 's needed they will prescribe them, so I tend to go on what they say with babies.’* (FG2, p1)

GP-led antibiotic stewardship efforts and education of specific knowledge at the point-of-care was important to participants.

*‘My first thought would be if a doctor says, “We can get through this without antibiotics”, then that would be my preference. I rely on their opinion.*’ (FG2, p3)

However, one parent noted that the differing opinions of doctors within practices can be confusing, emphasising the importance of consistency across healthcare points of contact with patients.

*‘It was just that conflicting advice of, if one doctor … [prescribes it] but then a second doctor [says] … they wouldn’t have prescribed it …*’ (FG2, p3)

Another mother's comment illustrated how a parent’s instinct about their child sits alongside, and possibly carries equal weight to, a GP’s advice, reinforcing the need to move beyond a more conventional telling or provision of advice by doctor to patient, to the holding of a constructive two-way exchange. A more nuanced dialogue around antibiotics use/AMR may better address any barriers to adopting a mindset where antibiotic use reflects concerns around AMR.

*‘For my son, I know him better than any doctor does.’* (FG2, p3)

Together with key theme 2 (socio-cultural influences on KABs), the doctor **–**patient consultation setting may be optimised with a more personalised approach to facilitating and addressing any barriers to appropriate antibiotic use/AMR.

## Discussion

A central concept underpinning this study is taking action at an individual level to mitigate the threat posed by AMR to society – projected at around 10 million deaths from AMR by 2050 if no action is taken (2016 UK AMR Review). However, evidence from this study suggests that, despite parents acknowledging AMR as a possible future problem for society (including for their child), the threat is not considered real or relevant enough to justify the sacrifice of their child foregoing antibiotics, and the immediate need to seek medical help/antibiotics is the overriding concern. This, in itself, is not entirely surprising, but it does serve to highlight that framing messages around antibiotics/AMR in terms of risks and benefits to their individual child now, versus risks and benefits to society in the future, may resonate more with parents and possibly the wider public too.

This trade-off between individual versus societal benefit is reflective of the dynamics that sometimes underpin attitudes towards COVID-19 vaccination, the benefit of which is often more apparent at a population level, especially in younger people (≤40–50 years) who are at lower risk of severe disease. At the time of the study, such vaccinations were not publicly available, but they were widely discussed, including risks and benefits to individuals and society. In effect, the COVID pandemic may have attuned collective thinking around the concept of individual versus societal benefit with regards to widescale health. Few members of today's UK population have previously had to make the personal sacrifices that they did during the pandemic, *e.g.* isolating or restricting socialising to primarily benefit population health. As remarked upon by participants, the early months of COVID also highlighted the lack of effective treatment, a scenario paralleled by attempts to manage a (multi-) drug-resistant infection.

Our study also reinforces that the fundamental misunderstandings relating to the biological basis of AMR persist among parents and may partially explain the finding that most parents do not perceive the relevance or benefit of harnessing a personal sense of responsibility towards mitigating AMR. ^8^^,^^31^

Finally, insights on the socio-cultural drivers of KABs around AMR are notable and suggest a more personalised doctor–patient dialogue around AMR may be more constructive. A 2018 WHO survey of antibiotic awareness campaigns showed that, to make further progress, campaigns should move towards locally adapted communication. Just as public health campaigns need to localise, so patient communication at the consultation level needs to be tailored to the individual. ^32^

Our data build on those already published on public perceptions of antibiotic use and AMR, ^8^^,^^31^ and will inform future research on how to enhance the relevance and impact of such communication for both society and individuals in their relationship with antibiotics.

### Comparison with existing literature

Addressing the topic of parental KABs around antibiotic use and AMR through the concept of individual agency to avert AMR is novel but notably builds on a growing body of relevant, associated literature.

Similar to our findings, another recent study observed that, ‘parents found it difficult to interpret symptoms and signs’, and, ‘[…]need better information and support to manage their child’s illness at home’, adding further emphasis to the continuing need for intervention in this respect. ^33^ Likewise, the perceived vulnerability of children, has been documented elsewhere. ^8^

Previous studies with families attest to long-held misunderstandings about AMR, for example, parents perceiving that they are at low risk because they infrequently use antibiotics, and that the 'body becomes immune to them [antibiotics]'. ^2^^,^^17^ Our study reinforces that such misunderstandings persist and suggests that more novel and potentially more effective means of engagement are needed.

A 2016 study noted the complexity of the doctor **–**patient interaction and 'interplay of care seeking'. ^34^ Our study reinforces and importantly builds on this by noting the nuances of parental background influences on KABs, suggesting that recognising, for example, ethnic, generational and country-of-origin differences may enhance the effectiveness of the doctor **–**patient dialogue.

Prior studies have referred to the benefits of the point-of-care setting to optimise AMR engagement, but more research is needed on how this may manifest in practice. ^12^^–^^14^^,^^17^^,^^35^^,^^36^ Pointedly, Cabral's 2016 study concluded the need for interventions that reduce antibiotic prescribing 'to address within-consultation communication, prescribing behaviour, and lay beliefs simultaneously'. ^35^ Our study advances this with specific aspects of 'how' these within-consultation communication and lay beliefs may be approached and leveraged to further shape mindset towards antibiotic use/AMR, for example, focus on the risks and benefits to the individual of judicious antibiotic use. It also suggests that making this within-consultation communication more real and relevant to the individual's immediate situation may be more impactful.

### Strengths and limitations

Participant recruitment comprised a broad cross-section of parents from South-East England, which provided a rich demographic diversity due to this area having a high population density, of widely variable backgrounds, across both urban and semi-rural settings. However, there may have been some selection bias, with most parents having very good levels of education and employment, which is largely unrepresentative of the general UK population.

Pandemic restrictions means the virtual (at-home) setting may be viewed as both a strength and a limitation. The format facilitated attendance by parents whose childcare commitments might have prevented attendance at an in-person group. However, this also led to some unavoidable parenting distractions.

Participant numbers were small, and it is not possible to draw generalised conclusions based on the comments of only 14 participants, but insights would draw strength in combination with findings from other studies, with our findings reinforcing and building upon prior findings.

The virtual format also meant the group was shorter in duration, and that the interactions between group members were less natural than an in-person group. In addition, resource constraints and the virtual format meant non-verbal group dynamics could not be recorded.

## Conclusions

These observations, in combination with a public engaged with the risks of infectious disease by the COVID-19 pandemic, suggest that clinicians and policymakers may frame messages around antibiotic use/AMR with an emphasis on the here and now (ideally delivered at the point-of-care), and drawing on relevance to their individual child at the present time, rather than referring to the impact of the future possibility of AMR for society. Tailor-making messages that are real and relevant to the individual would also benefit from a more nuanced approach that recognises the influence of an individual's multi-faceted socio-cultural background. Table 6 provides conclusions and recommendations based on this study.

**Table 6.** Conclusions and recommendations.

Conclusion	Recommendation
1. Parents feel their child’s immediate medical needs are more important than longer-term societal ones, and any distant threat of future AMR.	Frame AMR in the here and now, primarily on risk/benefit of antibiotic use/AMR for the individual. Clinicians should draw on nuanced socio-cultural influences that impact patient decision-making.
2. Parents feel that GP-led antibiotic stewardship and efforts to communicate around antibiotic use/AMR yield benefit and should be optimised.	Reinforce doctor-led, point-of-care discussions around AMR, during consultation with a symptomatic child.
3. Continuing need to educate on how antibiotics work, and on how AMR develops. Many parents misunderstand and think that AMR only affects people who overuse them.	Clarify any misunderstanding about antibiotic and AMR mechanisms at the point-of-care.
4. Lack of clarity among patients about when, and with which symptoms to seek professional medical help.	Provide clearer information on which symptoms and severity warrant medical help.
5. Measures to mitigate spread of coronavirus have primed public KABs around taking action now to prevent harm to wider society, *e.g.* vaccination.	Draw on the experience of vaccination and mitigation measures during the pandemic where people recognise a health threat if preventative actions are not taken in the present to preserve everyone’s future health.

Novel insights on how the pandemic has shaped participant views of infections, antibiotics and AMR illustrate how tapping into an individual's sense of immediate need including risks and benefits of antibiotics may better resonate (and possibly motivate too) with concepts such as foregoing an antibiotic to benefit both the individual and society, in a similar way to most individuals undergoing COVID vaccination to benefit not only themselves, but also, possibly more so, those people around them. Effectively, AMR messaging needs to leverage individual benefit to maximise societal gain. It may also draw on the concept that healthcare does not always have an effective treatment, as seen with management of COVID and with AMR.

Future research may investigate how to draw on these findings of individualisation and contextualisation, as well as timing and setting to both improve understanding and tailor meaningful communication that resonates with the parents of young children and the public more widely, to optimise parental sense of agency towards mitigating the threat of AMR.

## Ethical approval

NHS Health Research Authority (HRA) ethical approval (REC number 19/LO/1820, HRA approval February 2020.

## Consent

Written (electronic) informed consent for publication of the participants' details was obtained from the participants.

## Data Availability

Open Science Framework (OSF): Underlying data for ‘Sense of personal agency towards mitigating the threat of antibiotic resistance: a focus group study with parents of children under-5 years, conducted mid-pandemic’. https://doi.org/10.17605/OSF.IO/VN3X5. ^37^ The project contains the following underlying data: ED 1: Topic guide (full version). ED 2: Table of descriptions according to categories and codes. ED 3: Categories and their codes (and sub-codes). ED 4: Example of the framework matrix for category 4: Consequences of antibiotic resistance. ED 5: Study Protocol. ED 6: COREQ guidelines checklist Data are available under the terms of the Creative Commons Zero “No rights reserved” data waiver (CC0 1.0 Public domain dedication). First author: Becky McCall becky.mccall.18@ucl.ac.uk
