# Supplementary material for: Sense of personal agency towards mitigating the threat of antibiotic resistance: a focus group study with parents of children under 5 years old, conducted mid-pandemic
Source: F1000Res. 2025 Jun 10;11:1487. Originally published 2022 Dec 12. [Version 3] doi: 10.12688/f1000research.128550.3 (PMC12203309; doi:10.12688/f1000research.128550.3)
Supplement: Peer review history

# Reviewer report: Reviewer response for version 2

**DOI:** 10.5256/f1000research.178221.r387763

**Published:** 2025-06-23

**Authors:** Arne Simon

**Affiliations:** 1 Universitaetklinikum des Saarlandes Homburg, Homburg, Germany

Many thanks for this small but excellent qualitative study.

 As a paediatric infectious disease specialist who has dealt with these kinds of questions for more than 25 years, I would just like to add one thought. I do not understand how we can justify the decision against antibiotic treatment as a 'sacrifice' for the sake of future generations (to reduce the threat of antibiotic resistance). 

 This would only be the case if we decided not to treat a bacterial infection in a child with antibiotics, even though they definitely need them. 

 In reality, most children, particularly the younger ones, receive antibiotics without good reason due to diagnostic uncertainty, 'just for safety' or because the attending physicians complain that they do not have enough time to explain why antibiotics are not needed in this particular case. Freeing children from antibiotic overuse and injudicious antibiotic treatment is not a sacrifice, but a blessing.

 In this regard, I wonder how we (the experts) can come to the conclusion, that parents of infants should reject any antibiotic treatment, which is recommended by their family physician. As the authors stated, more should be done to explain the judicious use of antibiotics to parents, but the main problem remains at our side of the eqation.

 One Item in Tab. 2 is problematic: "White Jewish" is no ethnic origin. Jewish refers to a religion.

Is the work clearly and accurately presented and does it cite the current literature?

Yes

If applicable, is the statistical analysis and its interpretation appropriate?

Yes

Are all the source data underlying the results available to ensure full reproducibility?

No source data required

Is the study design appropriate and is the work technically sound?

Yes

Are the conclusions drawn adequately supported by the results?

Partly

Are sufficient details of methods and analysis provided to allow replication by others?

Yes

Reviewer Expertise:

Paediatric infectious dieases, antibiotic stewardship in paediaric patients.

I confirm that I have read this submission and believe that I have an appropriate level of expertise to confirm that it is of an acceptable scientific standard.

# Reviewer report: Reviewer response for version 2

**DOI:** 10.5256/f1000research.178221.r375581

**Published:** 2025-05-19

**Authors:** Daniel Czyz

**Affiliations:** 1 University of Florida, Gainesville, Florida, USA

The manuscript by McCall et al., entitled “Sense of personal agency towards mitigating the threat of antibiotic resistance: a focus group study with parents of children under 5 years old, conducted mid-pandemic” examines the **knowledge, attitudes, and behaviors of parents regarding antibiotic use and antimicrobial resistance (AMR)** **, **particularly for their young children. Conducted through **virtual focus groups with mothers** in England during the COVID-19 pandemic, the study explored their understanding of antibiotics and AMR, their **sense of personal responsibility in mitigating AMR** **, **and how the pandemic influenced their views on infection control. The findings indicate that parents often **prioritized their child's immediate health needs over the future societal threat of AMR** **.** The study highlights the **importance of tailored communication** between doctors and parents at the point of care to improve understanding and promote appropriate antibiotic use, suggesting that framing the message around individual benefits may be more effective. Addressing the following several major and minor comments will strengthen the publication.

**Major comments and recommendations:**

 -Several sentences and paragraphs are very consuming and unnecessarily complex. Please simplify. For example,

 - Prior studies have referred to the benefits of the point-of-care setting to optimise AMR engagement, but more research is needed on how this may manifest in practice.12–14,17,36,37 Pointedly, Cabral’s 2016 study concluded the need for interventions that reduce antibiotic prescribing ‘to address within-consultation communication, prescribing behaviour,

 and lay beliefs simultaneously’.36 Our study advances this with specific aspects of ‘how’ these within-consultation communication and lay beliefs may be approached and leveraged to further shape mindset towards antibiotic use/AMR, for example, focus on the risks and benefits to the individual of judicious antibiotic use. It also suggests that making this within-consultation communication more real and relevant to the individual’s immediate situation may be more impactful. – this whole paragraph is very confusing.

**-**“Participant numbers were small, and it is not possible to draw generalised conclusions based on the comments of only 14 participants, but insights would draw strength in combination with findings from other studies, with our findings reinforcing and building upon prior findings.” – also very confusing.

 - Using the term parents when only mothers were interviewed is incorrect. Parents should be changed to mothers.

 - “Alternative open access software to NVivo exist for example, Taguette or RDQA, but these were not used in this study.” - This information seems irrelevant and can be removed.

 Minor comments:

 -Side-oriented tables are difficult to read.

 -Table 2: under “Demographic characteristic (number participants)”, “Number of parents with children < or < and >5 years old(n)” should be < or < and ≥5 years old(n)

 -It would be helpful if the authors add the description of “thematic analysis” such as:  being a six-phase process:  familiarizing yourself with the data, coding, generating initial themes, reviewing themes, defining and naming themes, and producing the report.

 -Change GPpatient to “GP/patient” or GP-patient.” GP is also not defined. What is GP?

 -“A central concept underpinning this study is the taking of action (foregoing an antibiotic for a non-serious infection) at by an individual to mitigate the threat posed by AMR to society…” - Remove “at” after parentheses.

 - MS Teams – write out Microsoft

 - COVID-19 pandemic should be used throughout for consistency, not COVID.

 -“ This trade-off between individual versus societal benefit is reflective of the dynamics that sometimes underpin attitudes towards COVID-19 vaccination, the benefit of which is often more apparent at a population level, especially in younger people (≤40–50 years) who are at lower risk of severe disease.” – this needs a citation

 -The authors tend to directly quote cited literature. Please paraphrase rather than quote.

 -The manuscript mentions three times that BM is a mother of your children. This information is unnecessary repetitive.

 -“In the UK, where around 80% of antibiotic prescribing occurs in primary care.” is not a sentence

 On page 4, the word computer does not need to be capitalized. “Transcripts of the recorded discussions were entered into Computer-assisted qualitative data analysis software (CAQDAS, NVivo Version 12 (RRID:SCR_014802)) to organise the data.”

  

- Discussion: Paragraph 4, final sentence: please remove “so”

-Briefly explain what are SAGER guidelines and provide a reference.

 -“However, despite this progress, inappropriate use/prescribing of antibiotics persists, with data from 2012 to 2017 showing wide variation in antibiotic prescribing practices of UK primary care clinicians. For example, the rate of antibiotic prescribing varied between 77.4 and 350.3 per 1000 consultations, while the percentage of repeat antibiotic courses within 30 days ranged from 13.1% to 34.3%” Citation needed.

Is the work clearly and accurately presented and does it cite the current literature?

Yes

If applicable, is the statistical analysis and its interpretation appropriate?

Partly

Are all the source data underlying the results available to ensure full reproducibility?

Yes

Is the study design appropriate and is the work technically sound?

Yes

Are the conclusions drawn adequately supported by the results?

Yes

Are sufficient details of methods and analysis provided to allow replication by others?

Yes

Reviewer Expertise:

Antimicrobial resistance, non-traditional antimicrobial therapies, AMR education

I confirm that I have read this submission and believe that I have an appropriate level of expertise to state that I do not consider it to be of an acceptable scientific standard, for reasons outlined above.

# Reviewer report: Reviewer response for version 1

**DOI:** 10.5256/f1000research.141151.r360546

**Published:** 2025-02-06

**Authors:** Huong Vu

**Affiliations:** 1 Oxford University Clinical Research Unit, Hanoi, Vietnam

The study examines KABs in relation to antibiotic use and AMR among parents of children <5 y.o. and identifies the relevance of COVID-19 pandemic experience and impact in the issue of AMR, using qualitative research. The manuscript can be improved in the following aspects:

1. The suggestion to draw on the experience of vaccination and mitigation measures during COVID-19 pandemic to the issue to AMR is relevant. However, the authors need to discuss/ elaborate on further the potential challenges associated with the AMR issue in comparison with COVID-19. Particularly, AMR is a silent pandemic – unlike COVID-19, and the consequences of AMR on society appear to take a much longer time to occur. In addition, did the authors directly ask the participants if/how the lessons learnt from COVID-19 control could be applied to AMR? There seems to be a lack of direct reference to the participants’ discussion quoted in the manuscript. If this was not asked directly to the participants, but inferred from the authors’ point of view – this should be made clear.
2. Introduction: 2 ^nd^ paragraph - “antibiotic resistance” should be “AMR” for consistency throughout? 4 ^th^ paragraph – mentioning the “2022 data showing wide variation across practices”, please summarize what are the variations.
3. Introduction: Last paragraph – mentioning “This study aimed to obtain a snapshot of the perceptions and behaviours…”, here the authors mentioned “perceptions” instead of “knowledge, attitudes” as described in the rest of the document. Why such a difference? What were considered “perceptions” as compared to “knowledge, attitudes” in this study?
4. Methods: please indicate how many participants were recruited through snowball sampling and how this can influence the data collected; can snowball sampling generate a sample size with more similar participants with similar viewpoints and experience?
5. Methods: the authors mentioned “piloting by two parents”, who were they? Were they included in the study sample size? Were they included in the protocol approved by ethics committee?
6. Data analysis: the authors mentioned about “alternative open access software to NVivo exist for example, Taguette or RDQA”, did the authors also use these alternatives and what were the added values?
7. Data analysis: “data saturation point” was mentioned; but how this was determined? What did the authors do when “no new codes were generated”? Did this mean the sample size (individual participants and groups) was somehow influenced when the saturation point was reached? The sentence was not clear.
8. Results: Please specify clearly if the 2 participants dropped out and 1 other- “a medical doctor”, “conflicted”- included in the total sample size of 14 parents participating the 3 groups.
9. Table 3: The subthemes were sometimes not clear, it would be better if each of the subthemes were made with a clear meaning. I understand that this could be due to the fact that each subtheme was a summary of the content but in some cases these were not clear what the authors wanted to summarize specifically. Eg. “specific or broad spectrum”, “cultural shift (or not)”, “More pressing problems in the world”, “misuse – overuse”..., these were not clear, and some undefined words such as “ABX”.
10. Results: Use of the words such as “sometimes”, “some”, “most”, “relatively common”… should be more specific by specifying how many of the participants stating a particular result. There were only 14 participants, which makes it less convincing when using the above words when describing the results.
11. Discussion – 1 ^st^ sentence is not clear, and the reference of the 2016’s review is no longer updated, suggest to rewrite and use more updated references.

Is the work clearly and accurately presented and does it cite the current literature?

Partly

If applicable, is the statistical analysis and its interpretation appropriate?

Partly

Are all the source data underlying the results available to ensure full reproducibility?

Yes

Is the study design appropriate and is the work technically sound?

Yes

Are the conclusions drawn adequately supported by the results?

Yes

Are sufficient details of methods and analysis provided to allow replication by others?

Yes

Reviewer Expertise:

Antimicrobial stewardship, antimicrobial resistance, implementation science

I confirm that I have read this submission and believe that I have an appropriate level of expertise to confirm that it is of an acceptable scientific standard, however I have significant reservations, as outlined above.

# Response

**Published:** 2025-02-12

**Authors:** Becky McCall

**Affiliations:** University College London, UK

Point1.  I've added in comment about COVID-19 being an emergency pandemic versus AMR being a 'silent pandemic'. Participants were not directly asked about 'potential challenges associated with the AMR issue in comparison with COVID-19', it was inferred after responses to questions about their approach to infection prevention and control. This question was asked during the pandemic.

 2.“antibiotic resistance” should be “AMR” - an explanation has been given noting that this study refers to bacterial AMR and that this will be referred to as AMR going forward throughout the article.  Added data on inappropriate prescribing practices: '...with data from 2012 to 2017 showing wide variation in antibiotic prescribing practices of UK primary care clinicians. For example, ﻿the rate of antibiotic prescribing varied between 77.4 and 350.3 per 1000 consultations, while the percentage of repeat antibiotic courses within 30 days ranged from 13.1% to 34.3%. This  highlights that continued efforts to effectively counter inappropriate antibiotic prescribing are needed. ^7 ,^ ^8'^

 3. The terms perceptions, knowledge, attitude, and beliefs all relate to how individuals interpret and respond to a health issue e.g. AMR. Perceptions relate to subjective impressions and meanings including sensory and cognitive interpretations;  knowledge is the most different in that it refers to factual understanding (objective and information-based) of the issue e.g. AMR/antibiotics (I will add 'Knowledge' to the copy); attitude tends to relate to evaluation or emotional response to the issue -positive or negative so this is subjective and relates to affective interpretation; belief is more deeply held than an attitude and is cognitive but subjective in origin and usually based on personal, societal, or cultural interpretations. Perceptions is therefore an overarching term that approximates to attitudes and beliefs but by adding the work 'knowledge' to the copy it should clarify the reviewer comment. 

 4. Explanation about snowballing and its limitations has been added to the copy.

 5. Parents who piloted the questionnaire were sourced via the university hospital 's(UCL?UCLH) Patient and Public Involvement facility. Four previous patients some of whom were parents were included in designing and piloting the questionnaire. This was included in the ethics consent forms. Two other parents answered questions to help the author assess lay comprehension of the questions .These 2 were not included in the ethics form but their input was valuable. 

 6. Upon advice of qualitative researchers, I just mention others exist so as not appear promotional  with respect to one analysis software.

 7. Text added: 'This represented the data saturation i.e. no new concepts that made significant contributions were found in  the data and indicated a good point at which to terminate coding.'

 8. Clarified with text added to the copy that clarifies that data were analysed from 14 participants not including those people who dropped out - they did not participate in the actual focus group and therefore did not provide any data for analysis.

 9. Clarified in the text eg 'pressing' change to important;  simple examples given for misuse and overuse. See text. 

 10. Removed the  phrases and word 'relatively common ' and 'some'. Replaced with 'four'

 11. Updated. New reference added Antimicrobial Resistance Collaborators. Global burden of bacterial antimicrobial resistance in 2019: a systematic analysis. Lancet. 2022 Feb 12;399(10325):629-655. doi: 10.1016/S0140-6736(21)02724-0. Epub 2022 Jan 19. Erratum in: Lancet. 2022 Oct 1;400(10358):1102. doi: 10.1016/S0140-6736(21)02653-2. PMID: 35065702; PMCID: PMC8841637.

# Reviewer report: Reviewer response for version 1

**DOI:** 10.5256/f1000research.141151.r240493

**Published:** 2024-03-01

**Authors:** Ateya Megahed Ibrahim

**Affiliations:** 1 Prince Sattam Bin Abdulaziz University, Al-Kharj, Saudi Arabia

1. The study's sample size seems quite small, particularly considering the diversity of perspectives that could exist among parents of children under 5. Have you considered expanding the sample to capture a wider range of experiences and attitudes?

 2. While the study delves into parents' knowledge, attitudes, and behaviors regarding antibiotic use and AMR, it may benefit from a deeper exploration of the socio-economic factors influencing these attitudes. How do income levels, access to healthcare, and education impact parents' decisions regarding antibiotic use for their children?

 3. The focus groups were conducted mid-pandemic, which undoubtedly influenced participants' perspectives. However, the study could provide a more thorough analysis of how specific aspects of the pandemic (e.g., lockdowns, fear of COVID-19 transmission) shaped parents' views on antibiotic use and infection prevention.

 4. It's interesting that the study primarily focused on mothers from parenting networks. Given that caregiving responsibilities are often shared among parents, did the exclusion of fathers limit the breadth of perspectives represented in the study?

 5. The study mentions the Framework Method of analysis but lacks detail on how themes were identified and analyzed within this framework. Providing more transparency on the analytical process would strengthen the study's methodological rigor.

 6. While the study acknowledges the influence of socio-cultural background on parents' KABs, it could benefit from a deeper exploration of how cultural beliefs and practices intersect with attitudes towards antibiotic use and AMR. How do cultural perceptions of illness and healthcare providers shape parents' decisions in this context?

 7. The study highlights the need to engage parents in discussions about appropriate antibiotic use. However, it's unclear how these findings can be translated into actionable strategies for healthcare providers and policymakers. What specific interventions or educational initiatives could effectively address the gap between knowledge and behavior identified in the study?

 8. The study emphasizes the importance of individual agency in mitigating AMR. However, it could delve deeper into the structural barriers that may hinder parents' ability to make informed choices about antibiotic use, such as limited access to healthcare resources or systemic inequalities in healthcare delivery.

 9. The discussion around the pandemic's influence on parents' views lacks nuance. How did factors like media messaging, government guidelines, and personal experiences with COVID-19 shape parents' perceptions of infection prevention and antibiotic use?

 10. As the lead researcher is a mother of young children and a medical journalist, there may be inherent biases that influence the study's design and interpretation of results. How did the research team mitigate potential biases and ensure objectivity throughout the study process?

Is the work clearly and accurately presented and does it cite the current literature?

Partly

If applicable, is the statistical analysis and its interpretation appropriate?

Partly

Are all the source data underlying the results available to ensure full reproducibility?

Partly

Is the study design appropriate and is the work technically sound?

Partly

Are the conclusions drawn adequately supported by the results?

Partly

Are sufficient details of methods and analysis provided to allow replication by others?

Partly

Reviewer Expertise:

nursing

I confirm that I have read this submission and believe that I have an appropriate level of expertise to confirm that it is of an acceptable scientific standard, however I have significant reservations, as outlined above.

# Response

**Published:** 2025-02-12

**Authors:** Becky McCall

**Affiliations:** University College London, UK

1. In the ideal world, I agree it would be helpful to expand the sample size but this is not possible due to resources with this preliminary piece of work that will contribute towards a larger research project which forms the basis of a PhD project.

 2. Although I appreciate the usefulness of understanding the socio-economic factors influencing these attitudes this is not directly related to this study. 

 3. It is true that the study was conducted mid pandemic, and information about specific aspects of the pandemic would be interesting, however the focus of this study  was AMR not Covid and as a result, such specific analysis are not possible on this occasion.

 4. My study was open to both mothers and fathers but only mothers volunteered.  It would've been helpful to have had fathers view as well because I agree it would've added interesting diversity to the responses.

 5. Clarification and more detail added - please see copy. 

 6. Your question is interesting: How do cultural perceptions of illness and healthcare providers shape parents' decisions in this context? This study was not designed to explore cultural perceptions directly, other than if comments about cultural perceptions were made during the course of answering other questions. It is certainly something to be considered in future work.

 7.Great question - this directly addresses the next part of my research, which has looked at digital storytelling as a tool to help translate the finding of this paper (and others) into actionable strategies for healthcare providers and policymakers. Digital storytelling is a specific intervention but would need to be used alongside other engagement activities and interventions to change KABs with respect to AMR and antibiotic use.

 8. Again, your propose very interesting additions to the paper but to address what you propose, i.e.limited access to healthcare resources or systemic inequalities in healthcare delivery would require a further study that was powered to address these issues specifically. The study took a higher level view of parents perceptions of AMR.

 9.Again, you propose very interesting additions to the study and the paper but to address what you propose i.e. media messaging, government guidelines, and personal experiences with COVID-19 shape parents' perceptions of infection prevention and antibiotic use, would require a further study that was power to address these issues specifically. The study took a higher level view of parents perceptions of AMR.

 10. Biases were declared so readers are aware of my background and incorporate this into their interpretation of the data. I work alongside my supervisors and the topic guide and the questions included as well as the data were discussed with them.   Being a mother too can help to connect with the participants on the one hand, but on the other it may make me as the researcher more responsive to certain comments over others. Given that I have declared my biases, then I would ask the reader to allow for this in their interpretation of the paper. ideally, these data form one small part of a much larger body of literature about the parental perceptions of AMR. I would suggest this paper adds to this body of literature and a more complete picture would be obtained by a more expansive review of the existing literature including studies that use much larger sample sizes and indeed ask other questions such as those as you suggested above. 
